# Electric Field Encephalography as a Tool for Functional Brain Research: A Modeling Study

**DOI:** 10.1371/journal.pone.0067692

**Published:** 2013-07-03

**Authors:** Yury Petrov, Srinivas Sridhar

**Affiliations:** Northeastern University, Boston, Massachusetts, United States of America; Universitat Politecnica de Catalunya, Spain

## Abstract

We introduce the notion of Electric Field Encephalography (EFEG) based on measuring electric fields of the brain and demonstrate, using computer modeling, that given the appropriate electric field sensors this technique may have significant advantages over the current EEG technique. Unlike EEG, EFEG can be used to measure brain activity in a contactless and reference-free manner at significant distances from the head surface. Principal component analysis using simulated cortical sources demonstrated that electric field sensors positioned 3 cm away from the scalp and characterized by the same signal-to-noise ratio as EEG sensors provided the same number of uncorrelated signals as scalp EEG. When positioned on the scalp, EFEG sensors provided 2–3 times more uncorrelated signals. This significant increase in the number of uncorrelated signals can be used for more accurate assessment of brain states for non-invasive brain-computer interfaces and neurofeedback applications. It also may lead to major improvements in source localization precision. Source localization simulations for the spherical and Boundary Element Method (BEM) head models demonstrated that the localization errors are reduced two-fold when using electric fields instead of electric potentials. We have identified several techniques that could be adapted for the measurement of the electric field vector required for EFEG and anticipate that this study will stimulate new experimental approaches to utilize this new tool for functional brain research.

## Introduction

Electro-encephalography (EEG) and magneto-encephalography (MEG) are well-established modalities of studying brain signals. EEG samples electric potential across the scalp, while MEG samples magnetic field (usually, one its component) several centimeters from the head's surface. High-density implementations of these methods typically use 64–256 sensors covering the top half of the head. Several recent studies compared the two experimental techniques in terms of their spatial resolution and the amount of information they offer about the underlying brain activity. EEG and MEG were found similar in these respects [1]–[4] despite the fact that MEG is likely to be less affected by the skull's low conductivity than EEG [5], but also see [6]).

EEG and MEG provide somewhat different information on the brain's activity: MEG is sensitive to only tangential sources (with respect to the scalp surface), while EEG is sensitive to both radial and (less so) tangential sources [7]. Therefore, it is not surprising that recording both EEG and MEG signals can provide additional information on the brain's activity by increasing the effective number of independent signals recorded [4], [8]. Simply increasing the number of EEG and MEG sensors might not have the same effect due to a significant cross-talk between sensors. It is believed that bringing electrodes within 2 cm of each other or closer (∼500 sensors) does not further improve spatial resolution of either method [4].

Unlike electric potential, electric fields associated with brain activity are not generally studied because of the difficulty of measuring these weak signals. Furthermore, considering that the electric field is given by the negative gradient of the electric potential measured by EEG, one might wonder whether measuring the electric field instead of its potential could provide any new information at all. It has been shown that a potential defined on a surface has a unique continuation to another surface as long as one surface encloses the other and the volume bound by the two surfaces is source-free [9]. A potential defined uniquely on two arbitrarily close surfaces is sufficient to calculate the gradient of the potential uniquely. Hence, an electric field outside of the head's volume is uniquely determined by the potential distribution on the scalp, and EEG measurements can, in principle, be used to calculate electric fields instead of measuring them directly.

Although theoretically possible, such a calculation is difficult to implement for several practical reasons. First, in order to calculate gradients of the scalp potential, one needs a dense measurement of the potential distribution, which calls for an unconventionally large number of small-diameter electrodes separated by distances of 1 cm or less. The high-density EEG caps or nets currently in use have at most 256 electrodes. The electrodes are, typically, 1 cm in diameter and are separated from each other by 3 cm or more. This makes gradient calculation using EEG relatively imprecise. Furthermore, a common data processing step in high-density EEG is to discount one or more 'bad' electrodes due to the amount of noise. The resulting data gaps at the locations of the discounted electrodes further undermine the electric field calculation. Finally, measuring scalp potentials is not feasible in many situations due to poor quality of electric contacts between the electrodes and the scalp, e.g., for subjects with very thick hair or, conversely, lack of hair (due to scalp cornification). Apart from this, the quality of the electric contacts quickly deteriorates during physical activity due to sweating, and the lightest movement of the electrodes on the scalp produces overwhelming amounts of noise.

It is, thus, likely that dedicated electric field sensors could improve current EEG techniques. The sensors could be used by themselves or in combination with conventional EEG sensors. However, prior to investing time and money into research and development of adequate field sensors, one needs to estimate the promise of the new technique. This defines the rationale of the presented study. Herein, we model the distribution of electric fields near the scalp and evaluate the use of these fields for functional brain research, a method we term Electric Field Encephalography (EFEG). We demonstrate that electric fields generated by cortical sources are more focused than their associated potentials. This makes EFEG a more local measure of brain activity and offers significantly more uncorrelated signals than EEG, provided that sensors are located either on the scalp or close to it and are characterized by signal-to-noise ratio comparable to EEG sensors. This, in turn, results in major improvement to source localization precision.

Two head models were used to simulate electric fields of the brain in this study: an anisotropic 4-shell spherical head model and a 3-shell boundary element method (BEM) model (see the Materials and Methods section for details). The purpose of using these two models is to compare EFEG and EEG as realistically as possible. Either model has its own inherent limitations. The BEM model omits the highly conductive cerebro-spinal fluid (CSF) layer, because this layer is too thin at many locations to model it with sufficient precision. It also cannot model the radial-tangential anisotropy of the skull's conductivity due to the nature of the BEM method. The 4-shell spherical model, on the other hand, models the latter two head properties, but cannot be applied to a more realistic head shape. Taken together, the results for these two models provide stronger support for the EFEG and EEG comparisons made in our study than would either model alone.

## Results

We first discuss electric fields produced by a single current dipole positioned inside the brain shell of the spherical model. Then, the number of uncorrelated signals provided by electric field measurements (EFEG) and potential measurements (EEG) is evaluated by principal component analysis (PCA) for spherical and BEM head models. Finally, the quality of source localization is compared between EEG and EFEG methods.

### Single Dipole Electric Fields

Electric fields produced by a current dipole can be calculated analytically for an anisotropic spherical shell model [10]. In the case of a radially oriented dipole, maximal 10 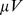 scalp potential (typical for human evoked potentials) is associated with ∼1 mV per meter maximal radial field component at the same location on the scalp. The maximal tangential component of the field is about 3 times weaker. In the case of a tangentially oriented dipole (with respect to the scalp surface), the maximal tangential component is about the same as for the radial dipole, while the radial component is approximately 3 times weaker than for the radial dipole. The corresponding potential and field scalp distributions are compared in Figures 3 and 4 in [10]. The dipole electric fields are characterized by significantly more focused distributions about the dipole location than those of the corresponding potentials. Hence, one might expect that there would be less cross-talk among nearby electric field sensors than for EEG sensors, and also that electric field measurements would be more indicative of the source location.

**Figure 1:**
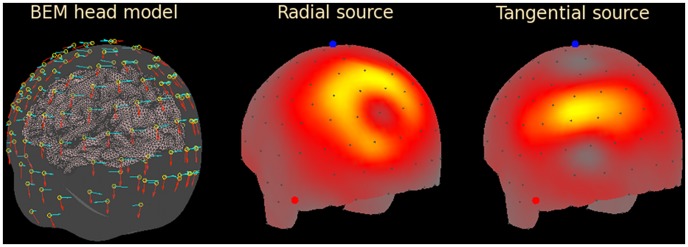
The BEM head model. Electric field sensor locations on the scalp and their orientations (red for inclination, cyan for azimuth) are shown in the left panel. Magnitude of the tangential component of the electric field on the scalp surface produced by (nearly) radial and tangential cortical sources are shown in the middle and right panels.

The electric field was also calculated for the anatomically more realistically shaped BEM head model as described in the Materials and Methods section. Scalp electric fields for two representative cortical sources are shown in Figure 1. The left panel illustrates positions of the electric field sensors on the scalp, with red arrows indicating sensors measuring inclinational component 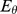, and green arrows indicating sensors measuring the azimuthal component 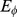. Tangential field magnitude 
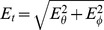
 is plotted in the next two panels using a colormap with hotter hues indicating larger field magnitudes. The middle panel shows 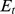 for one of the simulated 5124 source patches (see the Materials and Methods section) with dipoles oriented primarily in the radial direction. The right panel shows 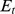 for a different source patch with dipoles oriented primarily in the tangential direction. 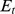 field patterns and magnitudes obtained for the BEM head model are similar to those patterns produced by single dipoles for the spherical head model (as compared with Figures 3 and 4 in [10]). In particular, radial and tangential sources produced characteristic ring-shaped and dumbbell-shaped patterns respectively.

### Number of Uncorrelated Signals

Given that electric field patterns are more focused than potential patterns, and that there are 3 times more electric field measurements than potential measurements (if all 3 field components are measured), one might expect that, in the practical case of a limited number of noisy sensors, the electric field would provide more information about brain sources than electric potential. The number of uncorrelated signals present in a set of data is one possible metric of this information. This metric can be calculated by modeling a multitude of realistic cortical sources, with signals measured by sensors located on the scalp or around the head, and then evaluating the degree of correlation among the resulting sensor signals. We simulated 5,124 source patches distributed evenly over the surface of left and right cortices (see the Materials and Methods section for details). The resulting potentials and electric fields were sampled by potential and field sensors positioned as discussed in the Materials and Methods section. Electric field components 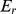 (radial), 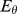 (inclinational), and 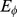 (azimuthal) at each sensor location were calculated for each source patch. Only tangential field components 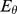 and 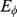 measured at the scalp surface were calculated for the BEM head model.

**Figure 2:**
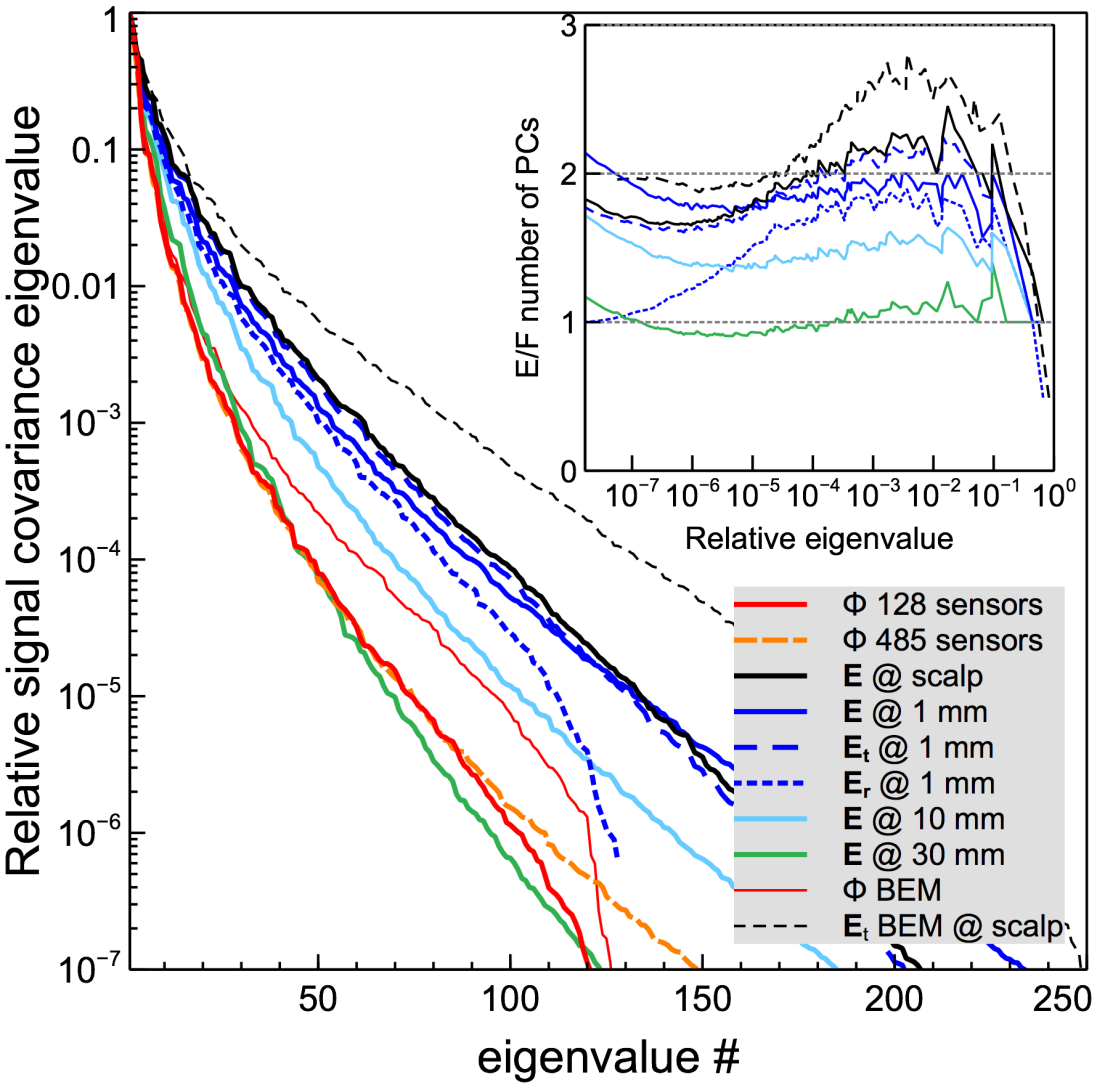
Normalized eigenvalues of the data covariance matrix plotted in the descending order. EFEG sensors at different distances above the scalp surface (0 mm/scalp, 1 mm, 10 mm, 30 mm) were simulated. The inset shows the ratio of the number of eigenvalues above a given value (plotted along the x-axis) between the electric field 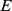 and the potential 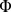 data.

To determine the number of uncorrelated signals the principal component analysis (PCA) was performed as described in the Materials and Methods section. The PCA eigenvalues normalized by the largest eigenvalue for each set are plotted in Figure 2 in descending order. The normalization allows one to consider the PCA eigenvalues in terms of the noise-to-signal ratio. Because PCA eigenvalues fall off very rapidly it is safe to assume that the overall signal power is of the same order of magnitude as the largest eigenvalue. Then, the number of eigenvalues (x-axis) above a given noise-to-signal power (y-axis) determines the number of uncorrelated detectable signals present in the data. PCA results for the scalp potential are plotted in red, results for the scalp electric field are plotted in black; for the electric field measured 1 mm, 10 mm, and 30 mm away from the scalp, the results are plotted in blue, cyan, and green respectively. Results for the BEM head model are shown by thin curves. One can see that electric field measurements provide significantly more detectable signals (eigenvalues above a given noise-to-signal value) than potential measurements for any noise level. This is indicated by the electric field curves lying above the potential (red) curves, with the exception of the green curve corresponding to EFEG sensors positioned 30 mm off the scalp. For example, for the noise-to-signal power 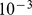 there were ∼30 uncorrelated potential signals vs. ∼60 (spherical model) and ∼90 (BEM) electric field signals.

To better illustrate the increase in the number of detectable signals, the ratio of the two curves for various noise-to-signal levels (y-axis values) was plotted in the inset to Figure 2. For the realistic range of noise-to-signal powers (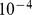
–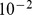) there were 2–3 times more usable signals in the field measurements than in the potential measurements when the field was measured on the scalp or 1 mm above it. For larger distances the advantage was quickly lost, so that at 30 mm the number of potential and field signals were about equal.

The increase in the number of uncorrelated signals did not occur simply because there were 3 times more field sensors than potential sensors. To show this, we simulated a 4-fold increase of the number of potential sensors by refining the 128-sensor mesh used for the rest of the simulations into 485-sensor mesh covering the same area of the head. The corresponding PCA results are plotted by the dashed orange curve. There was no significant increase in the number of usable signals except for unrealistically low noise levels (noise-to-signal power 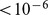).

It is also important to explain how different components of electric field contribute to the number of usable signals. To this end, PCA for EFEG sensors positioned 1 mm above the scalp surface was repeated for radial (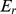) and tangential (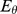 and 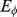) components separately using 128 and 256 sensors in each case. The results are illustrated in Figure 2 by the dotted and dashed blue curves respectively. The radial field alone provided almost two times more usable signals than the potential for the 1–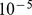 noise-to-signal power range. This agrees with the dipole radial field being more focused than the dipole potential [10]. Somewhat paradoxically, the tangential field provided more usable signals than the full field. As discussed below, this result appears less surprising once the sensor noise is considered.

### Source Localization

The analysis presented in the previous section shows that, assuming the same signal-to-noise ratio, high-density electric field measurements are more informative than potential measurements when taken near the head surface. Accordingly, a source localization algorithm should be able to reconstruct cortical sources more accurately when using electric field data. We calculated source reconstructions and associated localization errors for the same 5124 source patches used for the PCA. Details are given in the Materials and Methods section. Two source localization algorithms, MNE [11] and Harmony [12], were used for this purpose.

Both algorithms sought a ''distributed solution'', i.e., a pattern of activation distributed over thousands of cortical dipoles of fixed orientation and location. For a 'distributed solution' the number of usable signals is much smaller than the number of cortical dipoles, thus the inference problem is severely underdetermined, and some constraints are necessary to make the solution unique. The two algorithms use different constraints: MNE chooses the solution with the least power (activation), while Harmony, in addition, seeks a smooth solution. The rationale of the Harmony approach is that the high spatial frequency components of the solution cannot be reliably inferred from scalp data due to strong low-pass spatial filtering of the skull and high spatial frequency sensor noise.

**Figure 3:**
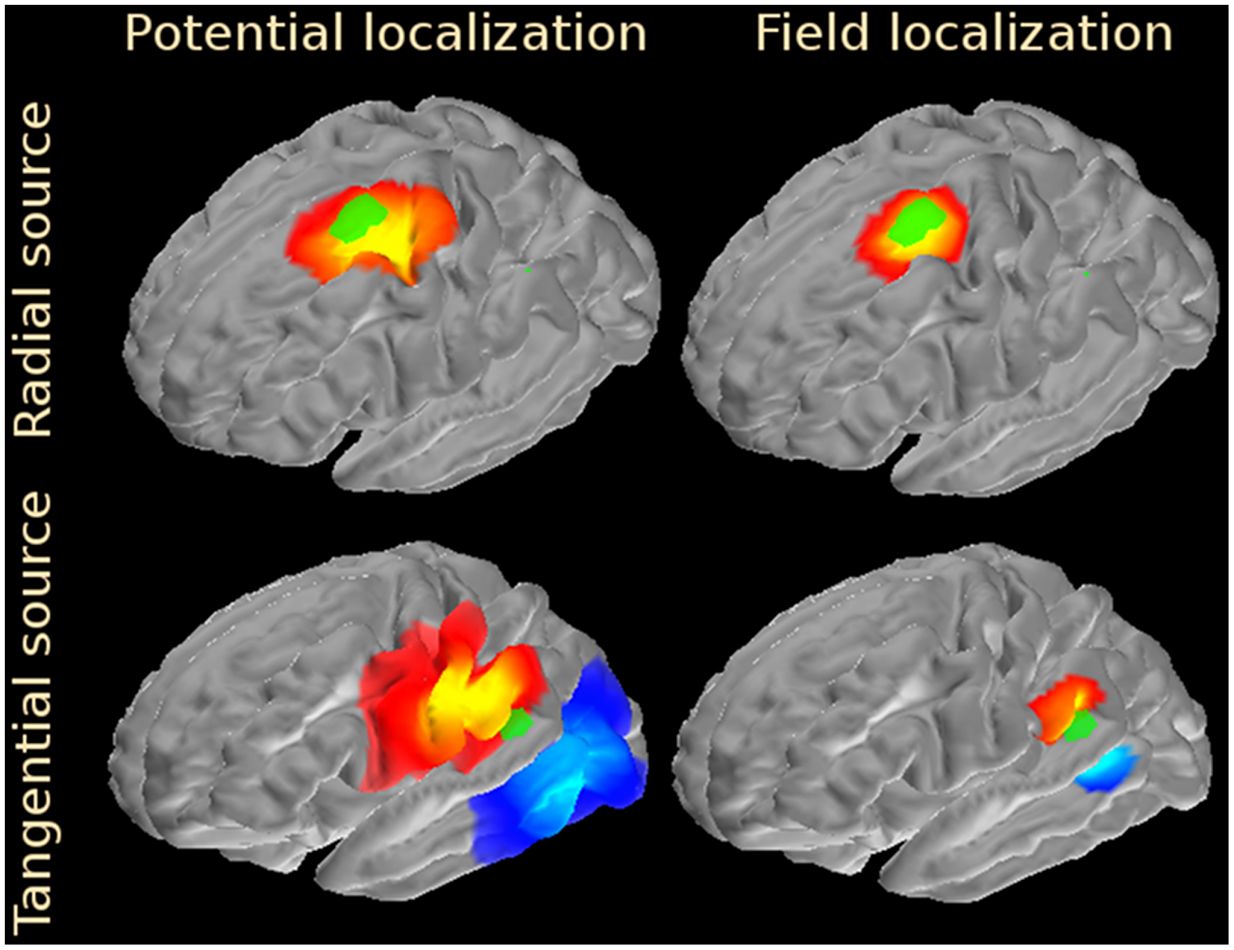
Representative reconstructions by the Harmony algorithm of two cortical sources. The results are shown on pial cortical surfaces, the 37-dipole source patch is shown by the green patch. Color indicates amplitude and direction of cortical currents: inward – cold, outward – hot.

Harmony BEM model source reconstructions for two representative source patches are shown in Figure 3. Results for radial and tangential sources are plotted in the top and bottom rows respectively. By tangential source we mean a source with tangential (parallel to the scalp) dipole component larger than radial (normal to the scalp) component. The panels in the left column display reconstructions based on potential measurements; the panels in the right column display reconstructions based on electric field measurements. Reconstructed cortical currents are shown by a colormap representing both magnitude and direction of the current dipoles: cold colors for inward currents, hot colors for outward currents. The currents were probability masked (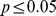, Bonferroni corrected using the number of sensors [13]). One can see that reconstructions based on the electric field measurements were tighter and located more closely to the simulated source patch shown as a green shape overlaid on the cortical surface. The improvements are particularly noticeable for the tangential source shown in the bottom row. Given that cortical normals are fairly randomly oriented, a simple calculation of solid angles shows that, proportionally, 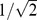 or about 70% of cortical sources are tangential.

**Figure 4:**
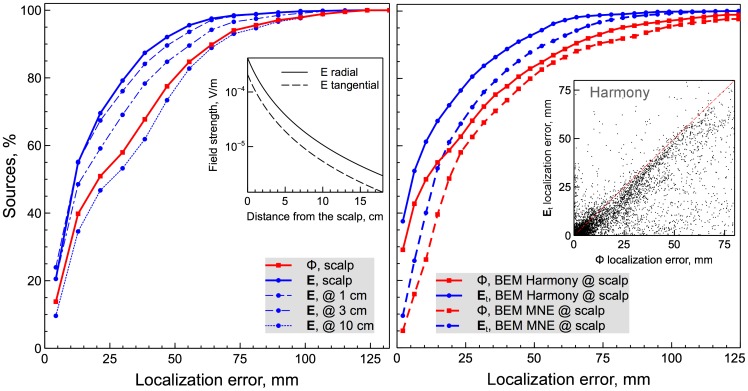
Source localization results for Harmony and MNE algorithms. The spherical model results are shown in the left panel, the BEM model results – in the right panel. The localization error, 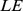, is plotted along the x-axis. The percentage of sources reconstructed with localization error smaller than a given x-value is plotted along the y-axis. Results for potential-based reconstructions are shown with squares, results for field-based reconstructions are shown with circles. Results for various electric field sensor distances from the scalp are plotted in the left panel using different curve styles. The inset shows the electric field fall-off with distance from the scalp. In the right panel BEM head model results for Harmony (solid curves) and MNE (dashed curves) algorithms are presented. The inset shows Harmony localization error comparison between electric field and potential localizations. Each dot represents one of the 5124 simulated source patches.

To quantify the localization error for all simulated sources the 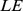 measure was calculated as described in the Materials and Methods section and the results were plotted in Figure 4. The source reconstruction results (Harmony algorithm) for the spherical head model are shown in the left panel. The 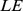 value is plotted along the x-axis, and the percentage of sources localized with a smaller error than the given x-value is plotted along the y-axis. Results for potential measurements are shown by the red curve, results for field measurements – by the blue curves. The field-based reconstructions were carried out for several sensor distances from the scalp: 0 (scalp), 1, 3, and 10 cm. In the figure, the corresponding results are traced with solid, dashed, dot-dashed, and dotted curves. Except for the largest distance (10 cm), all field-measurement curves are above the potential-measurement curve, indicating smaller localization errors for the field-based reconstructions even when there was a considerable separation of the field sensors from the scalp. It is important to keep in mind, however, that in these simulations the signal to noise ratio was kept constant for all sensor distances. For real measurements the signal-to-noise ratio will be decreasing due to electric field falloff with distance. This point is illustrated by the inset, where the maximal field strength for a radial dipole source located 1 cm below the skull is plotted as a function of the sensor distance from the scalp. Field strength decreases as the inverse of the cube of the distance for distances beyond 10 cm and even faster for smaller distances.

BEM head model results are shown in the right panel, where 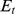 calculated at the scalp surface was used for electric field measurements. Harmony results are shown by solid curves, MNE results – by dashed curves. Similar to the spherical head model, field-based reconstructions had smaller localization errors for both Harmony and MNE algorithms. The inset shows a scatterplot of the Harmony localization errors (one dot for each of the 5124 simulated patches) comparing the potential-based localization error (x-axis) and the field-based localization error (y-axis). The majority of the dots fall below the diagonal indicating smaller localization errors for the field-based reconstructions.

## Discussion

In this study we introduced the notion of Electric Field Encephalography (EFEG) and used computer modeling to argue that measuring the electric field vector generated by brain activity has some significant differences from and advantages over, EEG. Electric field measurements can be conducted in a contact-free manner and, unlike EEG, where even the best choice of the reference point can contribute up to 15% of non-local signal [10], electric field measurements are truly local because they do not require any reference point. Our previous study reported formulas for calculating the electric field generated by a dipolar current source in a spherical model comprising concentric shells of anisotropic (radial-tangential) conductivity [10]. The formulas were used to show that scalp electric fields generated by a cortical current dipole were ∼1 mV per meter for 10 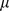
V scalp potentials. The electric field distribution over the scalp was shown to be more focal than the associated potential distribution.

The PCA carried out in the present study for spherical and BEM head models demonstrated that high-density measurements of electric field on or near the head surface can provide 2–3 times more uncorrelated signals of cortical activity than EEG measurements at the same locations (Figure 2). This can be explained by the more focused distribution of the electric field about the source location compared to the potential distribution. As a result, one can expect less cross-talk among sensors and more uncorrelated signals. The radial component of the electric field vector contained almost as many uncorrelated signals as the full field, which attests to a strong redundancy of the field components. As discussed in the Introduction, electric field in a current-free medium can be uniquely determined from the associated electric potential defined on a surface. Consequently, the field vector component normal to the surface can be determined from the two other components. The tangential components were somewhat more informative than the radial (normal to the scalp) components and even provided a slightly larger number of detectable signals than the full field for realistic signal-to-noise ratios. To understand this result it is important to stress that the PCA eigenvalues plotted in Figure 2 were normalized by the largest eigenvalue for each plot. Consequently, adding the radial field sensors increased the normalization constant as well as the rest of the eigenvalues. Assuming that experimental noise scales with overall signal, the noise scales with the largest signal's eigenvalue. Hence, in the framework of the presented PCA adding more sensors adds signals and also adds noise, which can effectively reduce some of the normalized eigenvalues. Given the field vector redundancy, the radial sensors apparently contributed more noise than signals, which explains the slight decrease of the normalized eigenvalues once the radial sensor measurements were included.

The largest increase in the number of uncorrelated signals between the potential and field measurements was observed for the BEM head model. There are several possible reasons for this: the BEM model fitted the brain more closely and, therefore, it had smaller dipole – sensor distances compared to the spherical model. The BEM head model produced less cortical current diffusion because it did not include the large gradient of conductance at the CSF – skull boundary. It also did not model the skull's anisotropy, which spreads currents still further within the skull [10]. In addition, deviations of the head shape from spherical symmetry make the simulated scalp currents more specific and, hence, more characteristic of the underlying cortical sources.

The increased number of detectable signals agrees with higher spatial resolution of bipolar EEG electrodes compared to conventional EEG electrodes [14], [15]. Provided that electrodes in a bipolar pair are sufficiently close-by, their measurement approximates an electric field's tangential component. A similar advantage exists for MEG sensors, where planar gradiometers analogous to bipolar EEG electrodes show superior spatial resolution compared to axial gradiometers [4]. Importantly, the signal-to-noise ratio is a critical issue for such bipolar sensors. In our analysis we assumed the same signal-to-noise ratio for EFEG and EEG. However, the potential difference across a closely spaced electrode pair is smaller than for the conventional EEG, and the resulting decrease in bipolar signal strength has to be matched by a proportional decrease in bipolar noise to obtain the same signal-to-noise ratio as for EEG.

The increased number of uncorrelated signals in the electric field measurements makes the EFEG approach potentially attractive whenever changes in brain state need to be interpreted based on real-time changes to its electric activity. Possible applications include noninvasive brain-computer interfaces, brain performance monitors, and neurofeedback. The increased number of uncorrelated signals should also improve source localization accuracy. Indeed, reconstructions of the simulated cortical sources were significantly improved when electric field measurements were used instead of electric potential measurements (Figures 3, 4). Localization errors for the Harmony algorithm dropped approximately two-fold between potential and field measurements for sources localized with better than 3 cm precision, which included about 2/3 of all sources. For the remaining sources, the improvement was less dramatic but still quite significant. The MNE algorithm showed qualitatively similar results, but its performance was poorer than Harmony's and its localization was improved less between potential and field measurements.

In principle, EFEG makes it possible to measure brain activity without making a physical contact with the scalp, once such electric field sensors become available. The contactless measurement has several advantages. It eliminates variability in electrode impedance (and consequently the amount of external noise), which is typical for EEG measurements, where electrodes have to be applied to the scalp and therefore such factors as hair, sweat, and skin state play a decisive role especially when electrodes cannot be readjusted during the measurement. Generally, EEG is very noisy for bald subjects due to cornification of the scalp, and also is very difficult to measure for subjects with thick curly hair. Electrode movement is another major factor impacting the quality of the signals measured with contact electrodes. This makes it very difficult to obtain high-quality EEG data for subjects performing tasks involving head movement.

We have identified several techniques that could be adapted for the measurement of the electric field vector required for EFEG, and anticipate that our study will stimulate new experimental approaches to utilize this novel mode of functional brain imaging. Several approaches to fiber optic sensors for electric fields have been demonstrated which could be used for EFEG [16]–[18]. These sensors appear to have sensitivity ∼1 mV per meter sufficient to measure the brain's electric fields, but a 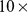 to 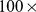 improvement in sensitivity would lead to more reliable observations of brain signals.

### Materials and Methods

Modeling of electric fields of the brain in this study was based on the electric field produced by a dipole current source positioned inside either a set of concentric spherical shells with homogenous anisotropic conductivity inside each shell (spherical head model), or inside a set of non-spherical shells with homogeneous isotropic conductivity (boundary-element head model, BEM). The electric fields for the spherical head model were calculated in [10]. Parameters of the spherical head model, BEM head model, and the simulation parameters used to estimate the number of uncorrelated brain signals and source localization errors are explained below.

### Spherical Head Model Parameters

**Figure 5:**
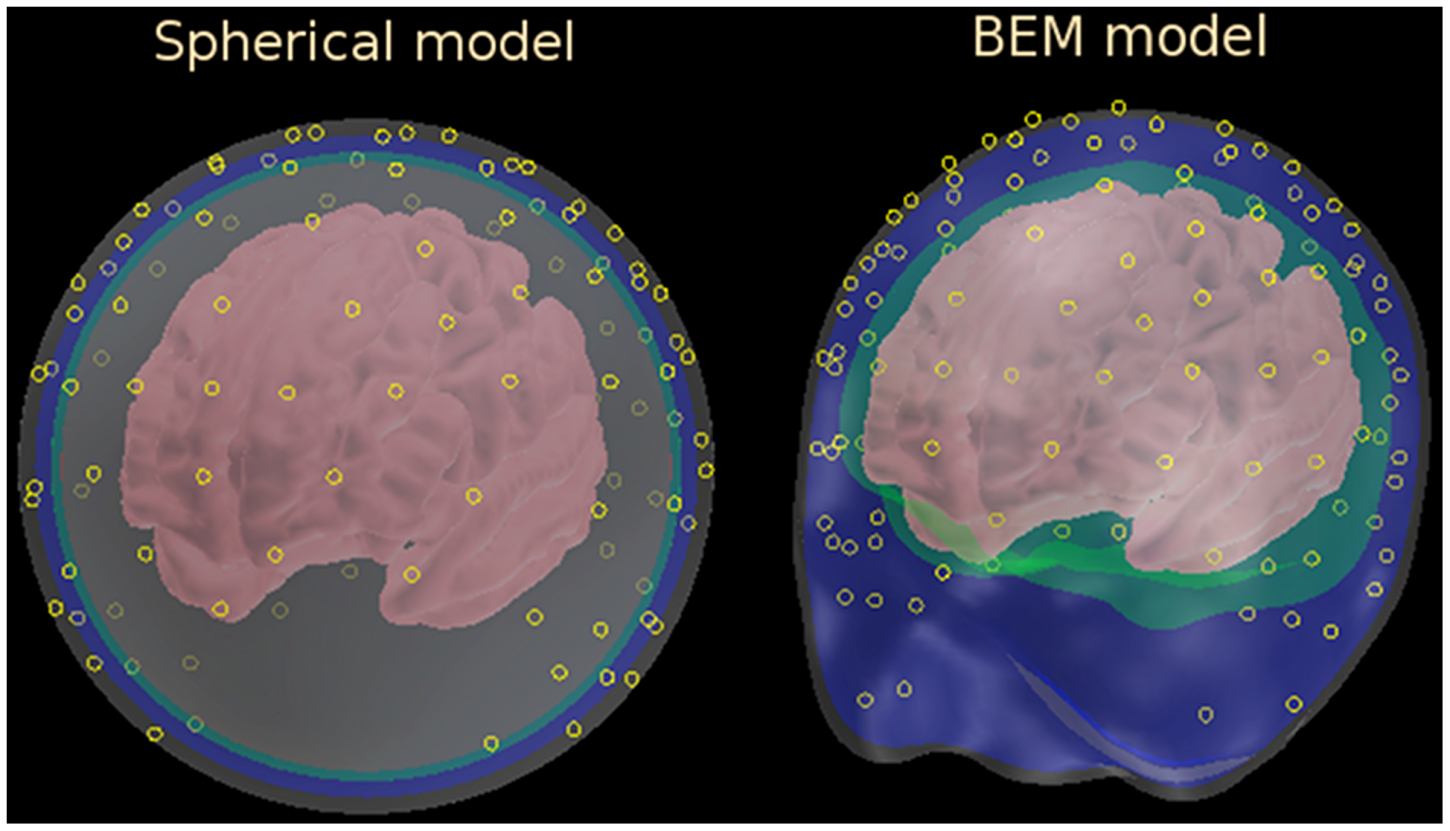
Spherical and BEM head models. Scalp, skull, CSF, and brain shells are shown in different colors from the outmost to the inmost shell respectively. BEM model makes no distinction between SCF and brain shells, both are shown in green. Pial cortical surfaces on which source dipoles were located are shown in pink. Yellow circles mark sensor locations based on 128-channel HydroCell GSN electrode net (EGI Inc.).

A 4-shell spherical model was used for simulations. The shells were (from innermost to outermost): brain (white and gray matter), cerebro-spinal fluid (CSF), skull (tables and diploë layers), and scalp (muscle, fat, and skin). We used spherical model parameters typical for human head tissue simulation. The outer radii of the brain, CSF, skull, and scalp shells were 9.1, 9.2, 9.7, and 10.2 cm respectively (Figure 5). The brain shell's radius (9 cm) was chosen to fit the averaged pial surface; this was used in simulations to determine current dipoles placement (FreeSurfer averaged brain, see the Sources section). The shells' radial conductivities were set to 0.3, 1.5, 0.006, and 0.3 S/m for the brain, CSF, skull, and scalp respectively. Tangential conductivities were equal to radial conductivities (isotropic conductivity) except for the skull shell, where the tangential conductivity was set to 0.06, i.e., a 10-fold anisotropy was assumed due to the higher conductivity of the diploë layer compared to the table layers of the skull. The values were based on the available anatomical data discussed in [19] and also in [2], [5], [20], [21]. As discussed in [10], [22] lower anisotropy ratios and higher skull conductivities might better describe a living human skull. For this reason, other skull conductivity parameters were tried as well (isotropic skull, 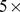 more conductive skull), but these manipulations did not change the results in any important ways.

### BEM Head Model

The boundary element method (BEM) head model was constructed based on high-resolution MRI data averaged over 40 subjects, the data were distributed as a part of the FreeSurfer toolbox [23]. The BEM head-model comprised the three volumes shown in Figure 5: scalp, skull, and CSF/brain; the volumes were reconstructed from the group-averaged MRI data with the help of the FSL toolbox [24]. The BEM solution to the Poisson's equation was calculated numerically using MNE Suite toolbox [25]. The tangential components of the electric field 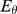 and 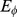 were obtained from the potential by numeric differentiation: the potential was calculated at triplets of points on the scalp, the first point in each triplet was at the location of one given sensor, while the other two were displaced from the electrode location by 1 mm in the azimuthal and inclinational directions respectively (Figure 1, left panel). The radial component of the electric field was not calculated. In order to improve the BEM's precision the inner skull was meshed as the 5-th subdivision of an icosahedron (20,480 triangles), the outer skull and scalp surfaces were meshed as the 4-th subdivision of an icosahedron (5,120 triangles). Conductivities of the three volumes were set to 0.3, 0.006, and 0.3 S/m for the scalp, skull and CSF/brain respectively. Because the BEM method can only be applied to isotropic volumes, skull anisotropy was not modeled.

### Cortical Sources

The cortical surface of the group-averaged data (provided with the FreeSurfer toolbox) was used to place current dipoles in simulations. 10,242 current dipoles were positioned at the nodes of a triangular mesh (5-th subdivision of an icosahedron, mid-gray FreeSurfer mesh) for left and right cortices. The neurologist's ''rule of thumb'' is that at least 6 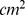 of cortex have to be active in order to record scalp potentials without averaging [19]. Correspondingly, a simulated source patch included a single dipole and all its nearest neighbors up to the third-degree coordination number, which gave 37 dipoles altogether: 1+6+12+18 = 37. The patches were roughly hexagonal in shape, approximately 2.5 cm in diameter, when measured along the cortical surface. This corresponded to 5 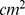 of cortical area, close to the “rule of thumb” size. The source patch was positioned at the nodes of a uniform grid (4-th subdivision of an icosahedron, 2,562 nodes) covering each hemisphere to simulate a total of 5,124 cortical activation sites.

The dipole orientations were fixed to be orthogonal to the cortical surface, reflective of the common assumption that EEG and MEG are due to synaptic currents produced by activity of cortical pyramidal cells. These currents flow along the pyramidal cells' axons, which are primarily perpendicular to the cortex. The magnitude of the source dipoles was equal among the 37 dipoles constituting a source patch and was such as to produce the maximum scalp potentials (for the most superficial sources) at about 10 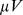, typical of human evoked potentials.

### Sensors

Realistic sensor locations were obtained by averaging a 128-channel HydroCell GSN net's (EGI Inc.) electrode locations, as they were applied to 34 subjects used for another study. The electrode locations were measured for each subject using a Polhemus FASTRACK digitizer. The locations were projected onto the spherical scalp for the spherical head model and slightly stretched and shifted by an affine transformation to better fit the scalp for the BEM head model. The resulting sensor locations are shown in Figure 5. In the case of the off-scalp locations for the spherical head model, sensors were shifted radially to the desired distance from the spherical scalp surface.

### Principal Component Analysis (PCA)

To determine the number of uncorrelated signals, the principal component analysis (PCA) was performed. First, a covariance matrix 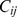 for the sampled data 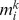 was calculated, with subscripts indexing sensors (128 for the potential, 384 for the field) and superscript indexing observations:
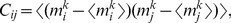
(1)the angular brackets denote averaging over 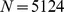 observations, 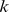, each observation corresponding to a given simulated source patch. Then, the eigenvalues of the covariance matrix were calculated.

### Source Localization

Two source localization algorithms, MNE [11] and Harmony [12], were compared in this study. In order to account for sensor noise and correlations among sensors, the source localization algorithms required an estimate of the noise-covariance matrix 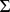. While such a matrix can be estimated from experimental data for potential (EEG) sensors no such data were available for electric field sensors. Instead, the simulated signal covariance matrix 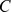, given by (1), regularized by a small amount of uncorrelated internal sensor noise, was used:
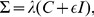
(2)where 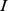 is the identity matrix,  is the regularization constant defining the amount of internal sensor noise, and 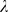 is the overall noise scaling parameter defining the solution's regularization. This choice of 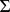 emulates noise from some (irrelevant) cortical activity as well as the internal sensor noise. The  value used corresponded to 3% signal-to-noise ratio for the internal sensor noise, the particular choice was not very important. The value of 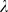 was more critical since it determines how well the solution fits the data versus how well the solution fits the constraints of the localization algorithm. 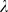 was chosen so as to minimize the mean localization error, separately for potential and field localizations, and for each of the two algorithms used.

The localization error was calculated as follows: first, the location of the true source was calculated by averaging the locations of its 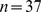 constituent dipoles; then, 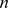 highest-amplitude dipoles were found in the solution for the same cortical hemisphere where the source was located. For each of the dipoles the distance 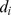 between the dipole 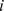 and the source location was calculated along the (pial) cortical surface. The raw localization error was then taken as the weighted average of 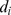:
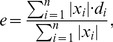
(3)where 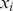 stands for the solution magnitude of the 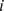-th dipole. Finally, the raw localization error was corrected for the extent of the true source 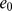, given by the above formula applied to the source patch itself:
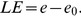
(4)

